# A Next Generation Sequencing-Based Protocol for Screening of Variants of Concern in Autism Spectrum Disorder

**DOI:** 10.3390/cells11010010

**Published:** 2021-12-21

**Authors:** Jie Huang, Jun Liu, Ruiyi Tian, Kevin Liu, Patrick Zhuang, Hannah Tayla Sherman, Christoph Budjan, Michelle Fong, Min-Seo Jeong, Xue-Jun Kong

**Affiliations:** 1 Athinoula A. Martinos Center for Biomedical Imaging, Massachusetts General Hospital, Harvard Medical School, Charlestown, MA 02129, USA; jhuang@bwh.harvard.edu (J.H.); jliu71@bwh.harvard.edu (J.L.); RTIAN2@mgh.harvard.edu (R.T.); kliu16@mgh.harvard.edu (K.L.); PZHUANG1@mgh.harvard.edu (P.Z.); htsherman@mgh.harvard.edu (H.T.S.); mfong@bwh.harvard.edu (M.F.); msjeong@mgh.harvard.edu (M.-S.J.); 2 Department of Global Health, Peking University School of Public Health, Beijing 100871, China; 3 Brigham Women’s Hospital, Harvard Medical School, Boston, MA 02115, USA; 4 Washington University in St. Louis, St. Louis, MO 63130, USA; 5 Dana Farber Cancer Institute, Harvard Medical School, Boston, MA 02215, USA; cbudjan@hms.harvard.edu; 6 Department of Psychiatry, Beth Israel Deaconess Medical Center, Harvard Medical School, Boston, MA 02215, USA

**Keywords:** Autism Spectrum Disorder (ASD), whole genome sequencing, whole exome sequencing, sanger sequencing, genetic report, trios, pathogenic variants, bioinformatics pipeline, precision medicine, molecular diagnostics

## Abstract

Autism spectrum disorder (ASD) is a neurodevelopmental disorder with strong genetic influences. There is an increasing demand for ASD genetic testing beyond the traditionally recommended microarray and syndromic autism testing; however, the current whole genome sequencing (WGS) and whole exome sequencing (WES) methods are lacking an academic standard for WGS variant annotation, reporting, and interpretation, tailored towards patients with ASD and offer very limited interpretation for clinical significance. Using WGS data from six family trios, we demonstrate the clinical feasibility and technical implementation of an evidence-based, fully transparent bioinformatics pipeline and report framework for an ASD-focused WGS genetic report. We confirmed a portion of the key variants with Sanger sequencing and provided interpretation with consideration of patients’ clinical symptoms and detailed literature review. Furthermore, we showed that identification of the genetic contributions of ASD core symptoms and comorbidities may promote a better understanding of the ASD pathophysiology, lead to early detection of associated comorbidities, and facilitate pharmacologic intervention based on pathological pathways inferred from the genetic information. We will make the bioinformatics pipeline and interpretation framework publicly available, in an easily accessible format, after validation with a larger cohort. We hope that the present proposed protocol can serve as a starting point to invite discourse and debate to further improve approaches in WGS-based genetic consultation for patients with ASD.

## 1. Introduction

The field of personal genomics is moving at an unprecedented pace, which is driven in part by reduction in the cost of next generation sequencing (NGS) technology and continuous expansion of databases correlating variants with clinical phenotypes [1]. Recent reports show that as many as 20% of participants in predispositional sequencing cohorts may have a variant with monogenic disease risk [2]. Moreover, there is a growing interest from the general public to understand the results of genetic testing in addition to having their genome analyzed.

Autism spectrum disorders (ASD) are neurodevelopmental disorders (NDDs) with typical features that include impaired communication and social interaction, as well as repetitive behaviors and restricted interests [3]. The prevalence of ASD is reported to be as high as 1 in 54 children in the US, with a male-to-female ratio of 4.3:1 [4]. Numerous studies support a strong genetic basis for ASD [5]. Based on clinical criteria, ASD can be broadly classified as syndromic or non-syndromic. Most individuals with an identifiable genetic cause can be stratified to a syndromic form of ASD, while the causes for the majority of idiopathic, non-syndromic ASD patients are more difficult to elucidate [6]. The pathogenesis of non-syndromic ASD is now thought to originate from complex interactions between environmental factors, such as environmental toxin exposure, prenatal infections, autoimmune conditions, as well as gut microbiome abnormalities, and genetic predispositions [6,7,8]. As of January 2021, the Simons Foundation Autism Research Initiative (SFARI) included a curated list of approximately 900 risk genes and less than 20 recurrent copy number variants (CNV) loci that are relevant to both syndromic and non-syndromic autism.

The American Academy of Pediatrics and the American College of Medical Genetics and Genomics both recommend chromosomal microarray (CMA), which is a technique that detects large duplications or deletions, as part of the first-tier evaluation for children with either a developmental disability or ASD [9]. If the CMA-based evaluations yield a negative result, the current guidelines recommend syndromic autism NGS panel testing. Nonetheless, the majority of such patients will not have any abnormalities detected on both assays.

The relative low yield with CMA and NGS panel testing is due to the vast majority of patients falling into the category of non-syndromic autism. The genetic contribution to non-syndromic autism continues to expand with the recent publications of large cohort case control studies [10,11,12]. Against this backdrop, genetic testing for patients with ASD beyond the official guideline recommendations is still controversial but has gained traction among clinicians and parents. Direct-to-consumer (DTC) labs, most of which lack expertise in autism or neurodevelopmental disorders, now offer whole genome sequencing (WGS), whole exome sequencing (WES), or single-nucleotide polymorphism (SNP) based genotyping, while some facilities offer expanded panels of autism risk genes beyond the syndromic variants. However, the gene lists included in commercial testing are inconsistent, incomplete, and are often outdated. For example, Hoang et al. found that the gene lists from 21 companies had only one gene in common, with only 12 companies that include one of the top autism risk genes CHD8 [13]. Recently, Shaaf et al. from the Hospital for Sick Children in Toronto, Canada summoned an international group of professionals to create a framework for combining all genes that have strong clinical ties to autism, which is the first step in providing meaningful interpretations of genetic tests for patients with ASD [14]. Based on our own clinical experience and published literature from recent studies, there is a rising demand for ASD genetic testing beyond the first-tier CMA panel testing [15,16]. However, many parents of individuals with ASD complain about the lack of transparency of analytical methodologies and are either confused by the DTC genetic testing results or unsatisfied with the way the genetic information is communicated, thus turning to their clinicians to seek a “second opinion” for genetic testing interpretation.

While the technical aspects of NGS variant discovery have matured significantly over the years, the creation of an NGS-based genetic report for complex disorders such as ASD require intricate knowledge of the disease pathophysiology, genetic underpinning, and significant clinical expertise. To our knowledge, there is no existing published academic standard for genetic test reporting using the WGS approach for patients with ASD. Building on the work of Shaaf et al., we attempt to create a framework for a transparent, evidence-based, and patient-centered ASD genetic testing and reporting pipeline [14]. We used a standard bioinformatics workflow for data processing and variant annotation and proposed a novel framework for reporting patients’ WGS results by prioritizing variants that have high “pre-test probability” of relevance to each patients’ clinical manifestations based on a comprehensive assessment of patients’ neuropsychiatric and comorbid conditions. The pipeline is based on the latest scientific evidence of genetic contribution of ASD pathogenesis from SFARI autism gene list, the ClinVAR database, and in silico protein functional prediction tools. Given the complex medical and psychiatric comorbid conditions in individuals with ASD, such as seizure, sleep disorders, gastrointestinal abnormalities, and immune dysfunction, the present report framework also aims to address potential genetic correlates with the patients’ comorbidities.

Although this article is not a research paper by convention, we are able to use the WGS data from six family trios to demonstrate the clinical feasibility and technical implementation of the bioinformatics and interpretation pipeline for an ASD-focused genetic report. We show that identification of the genetic contributions of ASD may promote the early detection and behavioral intervention of ASD, guide family planning, and facilitate pharmacologic-intervention trials based on pathological pathways inferred from both the patients’ variants and the dominant tissue/organ expression of affected genes [17,18].

## 2. Materials and Methods

### 2.1. Patient Enrollment

Data was collected in July 2019 from six unrelated proband-parent trios of ASD (4 males and 2 females) with ages ranging from 3–19 years old. Their diagnoses of ASD were based on the Diagnostic and Statistical Manual of Mental Disorders, Fifth Edition (DSM-5) and International Classification of Disease, 10th Revision (ICD-10). ASD severity was determined based on the Clinical Global Impression Scale (CGI-S) through evaluation by a clinician [19]. This study was conducted under ethical approval obtained from the institution’s Research Ethics committee and Internal Review Board (IRB) at Massachusetts General Hospital (IRB# 2017P001667 for blood draw, 13 July 2018; IRB# 2020P004102 for secondary data use and consent form approval, 7 January 2021). The study protocol and procedure of patient recruitment were conformed to the Declaration of Helsinki. All participants provided written informed consent to participate in the study as required by the IRB.

### 2.2. DNA Extraction and WGS

Peripheral blood samples from the affected child and parents were obtained at the Athinoula A. Martinos Center for Biomedical Imaging, Massachusetts General Hospital for DNA extraction. DNA extraction and library preparation were performed using standard protocol and WGS was performed by Novogene Co. Ltd. (Durham, NC, USA) on HiSeq X platform.

A total amount of 1.0 μg DNA per sample was used as input material for the DNA sample preparations. Sequencing libraries were generated using NEBNext^®^ DNA Library Prep Kit (New England BioLabs, Ipswich, MA, USA) following the manufacturer’s recommendations and indices were added to each sample. The genomic DNA is randomly fragmented to a size of 350 bp by shearing, then DNA fragments were end-polished, A-tailed, and ligated with the NEBNext adapter for Illumina sequencing, and further PCR enriched by P5 and indexed P7 oligos. The PCR products were purified (AMPure XP system; Beckman Coulter, Indianapolis, IN, USA) and resulted libraries were analyzed for size distribution by Agilent 2100 Bioanalyzer (Agilent Technologies, Santa Clara, CA, USA) and quantified using real-time PCR.

### 2.3. Sequencing Data Processing and Genetic Variant Discovery

The process of bioinformatics analysis of WGS data was broadly categorized into three steps: data processing, variant discovery, and variant annotation; we demonstrate the details of each step below. In brief, the original fluorescence images obtained from high-throughput sequencing platforms are transformed to short reads by base calling. These short reads (raw data) are recorded in FASTQ format, which contains the sequence information (reads) and corresponding sequencing quality information. Burrows–Wheeler Aligner (BWA) was utilized to map the paired-end clean reads to the human reference genome (hg38). The original mapping result in BAM format is further sorted by SAMtools, and Picard is utilized to mark duplicate reads and to deliver the final BAM files. The coverage and depth based on the final BAM file are then computed. The average sequencing depth for these 18 samples were shown in Figure S1. Most of these samples have an average sequencing depth above 30 for the 22 autosomes, which is standard for high-depth whole genome sequencing.

Genome Analysis Toolkit (GATK, version 4.1.6.0) was used for variant discovery. This process usually yields 3–5 million single nucleotide variants (SNVs), with an average sequencing depth greater than 30 and a coverage greater than 98%. Furthermore, GATK4 GerlimeCNVcaller was used to call CNVs. All the steps and parameters follow GATK recommended protocol.

### 2.4. Discovering Plausible ASD Associated Variants

**Figure 1:**
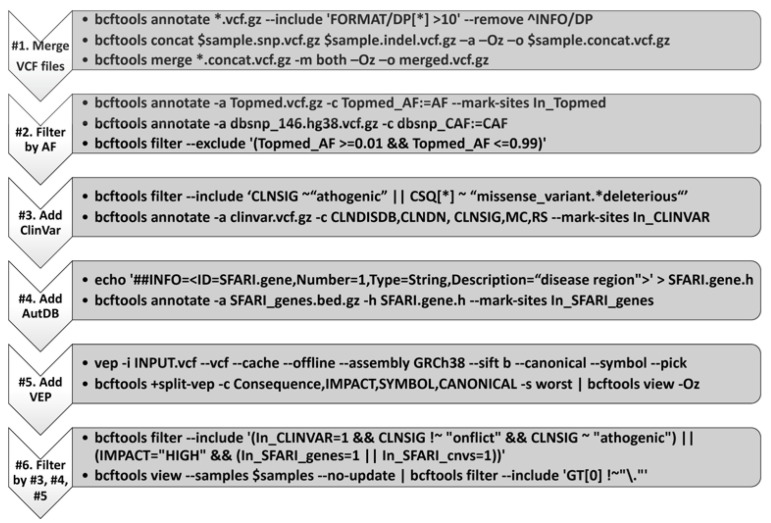
In-house variant annotation pipeline. VCF, variant call format; AF, allele frequency; ClinVar, a freely available, public archive of the relationships among human genetic variants and phenotypes (https://www.ncbi.nlm.nih.gov/clinvar/, accessed on 10 May 2020); VEP, ensemble variant effect predictor (ensemble release 105; https://uswest.ensembl.org/info/docs/tools/vep/index.html, accessed on 10 May 2020). Please note that a number following “#” refers to a step illustrated within the diagram.

**Figure 2:**
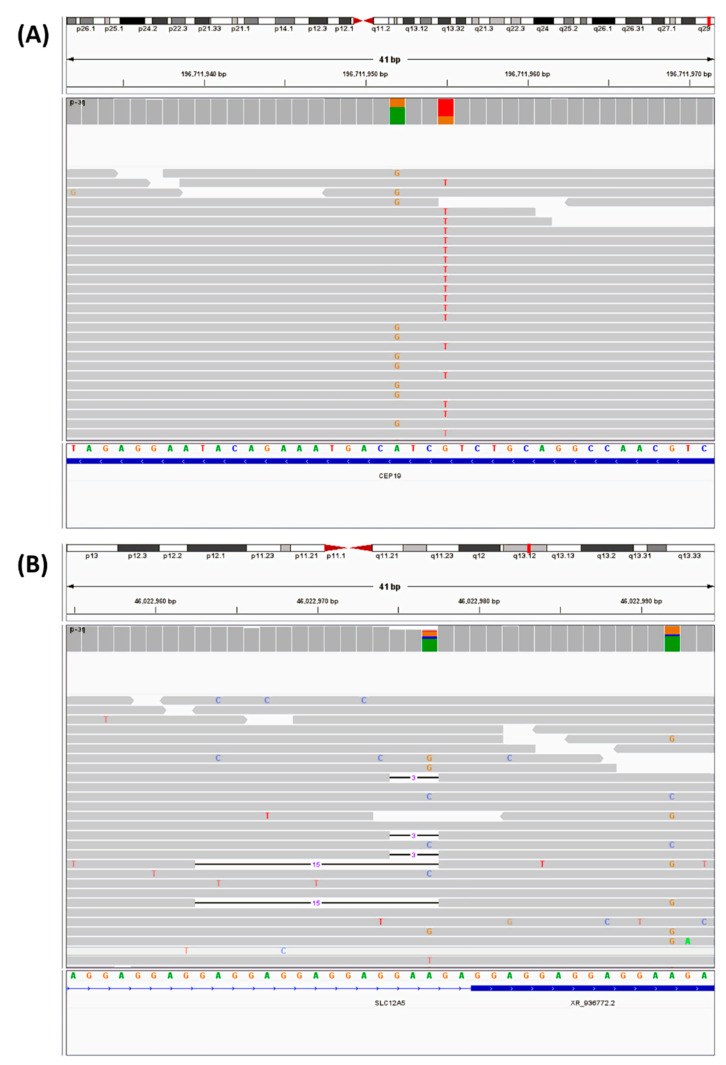
IGV view of two example variants identified by the bioinformatics pipeline. (**A**) A single nucleotide variation on the CEP19 gene of chromosome 3. (**B**) A potentially complex variation on the SLC12A5 gene of chromosome 20. This region was shown to have two in-frame deletion variants, but subsequent manual review found another de novo A:G single nucleotide substitution variant at base pair 46022977, which is confirmed by Sanger sequencing. The two in-frame deletions are not confirmed and most likely represent sequencing artifacts.

The above process generated a VCF file with about 5 million genetic variants for each subject. The process of identifying a few plausible genetic variants that are associated with ASD of the proband is similar to “picking a needle from a haystack.” We adopted an approach that weighed all evidence from population genetics, biological function, and clinical knowledge. The 6 steps are summarized in Figure 1 below. First, we created a single consolidated VCF file for each subject. Second, we added population frequency of all genetic variants and removed those that are common in the population (Topmed and GNOMAD, minor allele frequency (MAF) > 1%). Third, we added ClinVAR annotations for the variants that passed MAF filtering. Fourth, we added annotations from ASD databases AutDB and SFARI. ClinGEN was not used because the autism risk genes collected in the ClinGEN database are nested in the SFARI and AutDB gene list. Fifth, we used the VEP software to add the bioinformatics predicted annotation. Finally, we applied various filters based on the various annotations from the added annotations mentioned above. All variants discovered by the bioinformatics pipeline are subject to manual review on the Integrated Genome Browser (IGV) by staff members trained in human genetics and molecular diagnostics (Figure 2). Through this “cross-check” mechanism, we were able to identify variants that may be incorrectly called by the pipeline. In order for users to easily adopt our proposed protocol, we incorporated widely used basic command tools (mostly a single bcftools command line) rather than complicated third-party “black-box” algorithms.

### 2.5. Manual Review of Known ASD-Associated Variants

Although the bioinformatics pipeline for variant discovery has been thoroughly reviewed by multiple bioinformatics experts, we decided to implement an additional layer of “safety mechanism” to manually review a list of top genes or chromosomal duplication/deletion with known contribution to syndromic and non-syndromic ASD pathophysiology for each patient (Table 1). This approach was intended to increase our sensitivity for detecting abnormalities within the genes with highest risk and was inspired by the standard operating protocol/bioinformatic pipeline for Oncopanel, which is a targeted panel to identify cancer risk genes developed by the Brigham and Women’s Hospital and Dana Farber Cancer institute [20]. The gene list is generated via both in-house staff literature review using a framework similar to what is described by Shaaf et al. [14]. Individual patient’s raw sequencing data were reviewed on either the Varsome genome browsing platform or IGV.

### 2.6. Sanger Sequencing

We considered the scalability of our reporting framework from a cost-effectiveness perspective and only performed confirmatory Sanger sequencing on variants that either require further validation based on sequencing quality or on those that involve complex genomic regions. Take the four variants of concern discovered for patient #1 for example; for the SLC12A5 variant on Chromosome 20, the VCF file shows two independent structural variants with starting positions that are three base-pair apart, while the IGV plot demonstrates a variant with the characteristics of a frameshift. Therefore, we applied sequencing for this region and confirmed that the identified SLC12A5 variant in patient #1 was a de novo mutation. Specifically, Sanger sequencing confirmed the single nucleotide substitution (A to G mutation) in the proband that is not present in either parent. Based on feedback from ASD clinicians and researchers, we propose a variant prioritization scheme to select variants based on the pre-test probability of their relevance to individual patients’ pathophysiology. The following factors are considered: (1) whether the gene has known expression in the central nervous system (CNS), (2) whether its function has been linked to ASD pathophysiology based on the literature, (3) whether the variant is a known pathogenic or likely pathogenic mutation based on ClinVar annotation, and (4) whether reported conditions associated with mutations in a particular gene (either medical comorbidities or neuropsychiatric symptoms) have any overlap with a given patient’s clinical presentations. Due to the consideration of these factors, the selection of variants is a highly manual and individualized process for each patient. Extraction and purification of DNA from patient and parental samples was performed using Monarch Genomic DNA Purification Kit (NEB). PCR and sequencing primers were designed using Primer3 (https://bioinfo.ut.ee/primer3-0.4.0/) and Sanger sequencing was performed by Genewiz using standard procedures. The Sanger sequencing primer information is detailed in Table S1. In short, the DNA sample is divided into four separate sequencing reactions, containing all four of the standard deoxynucleotides (dATP, dGTP, dCTP and dTTP) and the DNA polymerase. To each reaction is added only one of the four dideoxynucleotides (ddATP, ddGTP, ddCTP, or ddTTP), while the other added nucleotides are ordinary ones. Following rounds of template DNA extension from the bound primer, the resulting DNA fragments are heat denatured and separated by size using capillary electrophoresis. The Sanger analysis consists of (1) ensuring that there is no issue with sequencing quality, (2) visualization of the sequences by ApE software (v3.0.8, Madera, CA, USA), which is an open source sequence visualizer/plasmid editor software commonly used in the molecular biology community (https://jorgensen.biology.utah.edu/wayned/ape/), (3) the sequences are compared with reference genome sequences in the same region manually to determine if mutations are present by Sanger; (4) mutations identified by Sanger are cross-compared manually with mutations identified by NGS.

## 3. Results

### 3.1. Patient Cohort Summary

A total of 6 ASD-parent trios were included in this case series (Table 2). Probands and both parents participated in this genetic testing process. Only probands were diagnosed with ASD. The probands vary in ASD functionality and comorbidities as summarized in Table 2. The cohort only consisted of patients of Asian ethnicity.

### 3.2. Summary of Variants and Sanger Confirmation

On average, our pipeline identified approximately four variants with potential functional significance in each patient’s WGS sample (Table 3 and Table A1). Among all the variants identified, 78.9% (15/19) are small-scale variants (e.g., SNVs, small indels), and 21.1% (4/19) are large-scale variants (i.e., duplications or deletions of chromosome segments). Based on our Sanger sequencing results, the majority of confirmed variants are of either paternal or maternal origins, with only two de novo mutations that were not present in either parent (Appendix A and Table A2). Interestingly, many of the variants are associated with ion channels, including *SLC12A5*, *SLCO1B1*, *SLC7A14*, and *GJB2*, which is consistent with current research that shows chanopathies are an important pathogenic driver of ASD [21]. Additionally, we applied this pipeline to the sequencing data of an ASD trio who visited the clinic in November 2021 and quickly identified a pathogenic variant within the PAH gene. This variant is maternally inherited. It is designated as “reviewed by expert panel” by ClinVAR, which is the highest level of confidence. The PAH gene is also listed in the SFARI database for association with ASD. Further testing and validation of our protocol in larger cohorts of ASD patients (e.g., Autism Speaks MSSNG project) is justified and is being actively pursued.

Among the 13 variants submitted for Sanger sequencing, nine (9/13, 69%) variants were confirmed, showing moderate concordance between Sanger and NGS. Sanger sequencing confirmed the presence of *SLC12A5*, *PROP1*, *CYP11B1*, *MYOC*, *SLC7A14*, *TXNL4A*, *SERPINB7*, and *GJB2* variants in the six subjects’ genomes. In addition, Sanger sequencing elucidated some deviations from the expected DNA variant calls based on NGS. Sanger sequencing failed to confirm *AIFM1*, *SLCO1B1*, *BSCL2*, and *PROKR2* variants in the patients’ genome.

### 3.3. Clinical Synopsis of the Case Series Illustrates the Utility of the Whole Genome Sequencing Test in Understanding ASD Pathophysiology and Comorbidities, Providing Targeted Treatment, and Offering Preventative Guidance

Patient #1 is a six-year-old girl who showed severe autistic symptoms with very limited verbal ability. She was identified with a *SLC12A5* (encoding KCC2) splice site variant which was confirmed by Sanger sequencing. The action of KCC2 is crucial for the inhibitory effects of GABA and glycine in most synaptic circuits and it is expressed predominantly in the CNS [22]. Frameshift mutations are relatively rare and are predicted to lead to a loss of function. Abnormal or decreased function of KCC2 causes neuronal excitation/inhibition imbalance (E/I) and is thought to contribute to the core symptoms of autism and seizure comorbidities [23]. Bumetanide is a NKCC1 blocker that decreases intracellular chloride concentration (achieving the same function as a KCC2 activator). Multiple clinical trials have demonstrated efficacy of alleviating core symptoms, at least in a subset of patients; it has been used in the clinical setting as an off-label adjunct treatment for patients with ASD [24,25]. Clinical studies show that it is generally well-tolerated in the pediatric population and the medicine is already approved by the FDA for the treatment of edema associated with congestive heart failure, and hepatic and renal diseases, including nephrotic syndrome. In addition, recent data suggests that KCC2 acts as a key target of oxytocin in postnatal events of GABA switch that may be linked to the pathogenesis of neurodevelopmental disorders. We provide visual displays of pathophysiology for all the variants that are relevant to ASD core symptoms and key comorbidities. Based on these findings, we recommended the patient to initiate oxytocin nasal spray and oral Bumetanide treatment in addition to the standard management program, in consultation with the patient’s primary psychiatrist and neurologist. In addition, this variant may also confer an increased risk for seizure. Although this patient has no history of seizure nor an abnormal EEG, we offered the advice of precaution and suggested a periodic neurology follow up.

Patient #2 is an eighteen-year-old male who has severe ASD. He was identified with a *GJB2* variant (confirmed by Sanger sequencing), which encodes the gap junction protein Connexin 26 and is expressed in many tissues. The identified *GJB2* variant has been most commonly associated with sensorineural hearing loss. Connexin 26 is also involved in calcium signal pathway and neuronal migration and some patients with *GJB2* variants have been observed to have seizure symptoms [26]. In addition, he was found to have a *PROKR2* variant; however, it was not confirmed by Sanger sequencing. The *PROKR2* gene also marks an increased risk for seizure. This patient developed seizures for the first time a year after this genetic testing. We warned the family about the possible risk of seizure when we delivered the report and offered consultation, indicating the value of the genetic testing for preventative guidance and early detection of comorbidities. Given the association between the *GJB2* variant and hearing loss, we also recommended patient to discuss with his primary physician to undergo a thorough auditory exam to assess for the potential roles of sensorineural hearing impairment as a contributor to the patient’s non-verbality and the use of cochlear implant if hearing loss is found.

Patient #3 is a nineteen-year-old girl with moderately severe ASD and generalized anxiety disorder. She was identified with *PROP1* and *CYP11B1* variants, both of which were confirmed by Sanger sequencing. *PROP1* encodes for the Paired-like homeodomain transcription factor located in the developing pituitary gland. *CYP11B1* encodes 11-beta-hydroxylase, which is essential for the synthesis of cortisol from the adrenal gland. Impaired adrenal cortisol/epinephrine/norepinephrine axis can impair social functioning. *PROP1* regulates pituitary hormone synthesis, which also regulates adrenal function. Dysfunction in the hypothalamus-pituitary axis can affect ASD core symptoms and explain the patient’s comorbid anxiety. As a result, a thorough endocrine workup was recommended to the family. In addition, given that the clinical symptoms of severe anxiety and variants in the pituitary-adrenal axis, we recommended the patient’s family to speak with her primary physician to consider beta blockade as well as vagal nerve stimulation as adjunct treatment.

Patient #4 with mildly severe ASD was identified with a variant in *SLC7A14*, an amino acid transporter highly expressed in the CNS lysosome and implicated in retinitis pigmentosa. Given its role in amino acid metabolism/recycling and the abundances of neurotransmitters with amino acid precursors, we recommended therapies that target the potential E/I balance [27]. The other two patients did not show variants with implications for ASD pathophysiology or comorbidities, suggesting that environmental factors may play a more prominent role in their pathogenesis.

### 3.4. Genetic Report Design: Summary of Variant Information and Clinical Interpretation in the Patient Cohort

**Figure 3:**
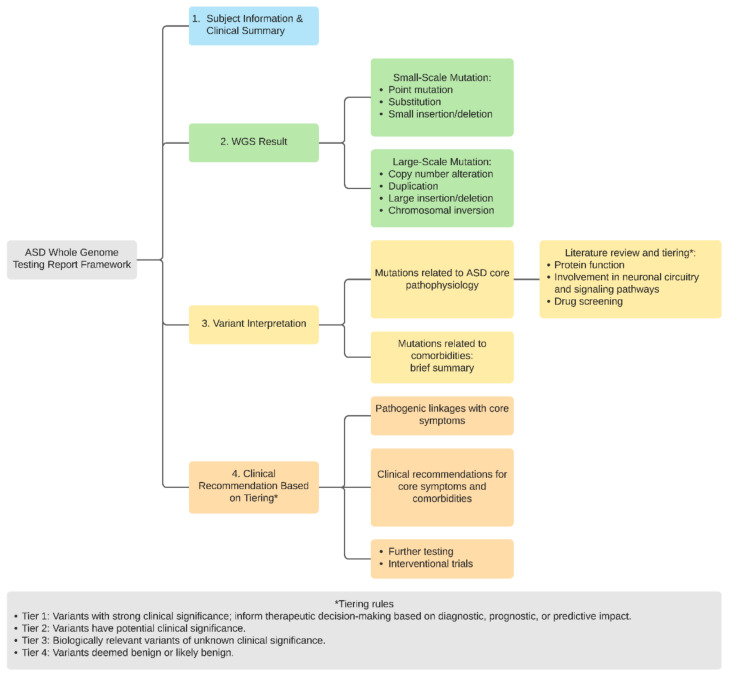
A summary of structure and components for ASD WGS genetic report.

We propose a genetic report framework tailored to patients with ASD that contains evidence-based results with the goal to provide a detailed explanation and actionable guidance to patients (Figure 3). The report consists of two major components: variant summary and clinical interpretation. The WGS result summary section highlights the precise variants (both small and large scale), variant types, parental origin of the variant (or de novo variants), relevance to ASD pathogenesis, and other medical conditions known to be associated with the variant.

Subsequently, a thorough review of evidence based on existing literature is provided for each variant (e.g., encoded protein function, tissue/organ expression pattern of the transcripts or protein, or any key in vitro/animal/human studies, biological pathways involved) based on literature and well-established open-source pathway mapping tools such as KEGG. Furthermore, we query FDA and pharmacological databases to elucidate whether certain variants or dysfunction in encoded proteins may confer candidacy to available medications that are either FDA approved or are in clinical trial. A diagram summarizing the key information is created for each variant (Figure 3). The next major component of the report consists of a clinical impression that attempts to address potential pathogenic contributions of each patient’s identified variants to autism core symptoms and other comorbidities. In addition, we referenced the ACMG list of incidental findings to highlight variants that belong to this list [28]. We adopt the framework of a four-tiered reporting system that is widely used for cancer somatic genetic testing [29] which is illustrated in Figure 3. Additionally, a sample report is included (Figure S2).

## 4. Discussion

In this paper, we systematically demonstrated the technical implementation and clinical feasibility of a WGS-based genetic report using six autism patient-parent trios as a case study. We created an evidence-based pipeline for sequencing data processing and genetic variant discovery using standard protocols coupled with Sanger sequencing confirmation. Subsequently, extensive data mining and literature review allowed us to predict the functions of the affected genes, their potential relevance to the patients’ disease and availability of targeted treatments. All these information is subsequently integrated into the genetic report. Although many of the variants may not qualify as “pathogenic” based on the ClinVar database, we are currently limited by the paucity of large, well-powered ASD genetic studies to precisely determine the risks conferred by variants in these genes that may have important functions in the CNS or affect systemic comorbidities that interact with ASD core symptoms. Therefore, the interpretation of the mutations depends largely on the pre-test probability as outlined before by considering the correlation between affected genes and each patient’s unique spectrum of neuropsychiatric symptoms and medical comorbidities.

In this clinically oriented framework, we thoroughly reviewed and explained the potential relationship and possible contribution of the genetic variants to the individual patient’s phenotypes, including core ASD symptoms and the co-morbidities, by using a tiered system. We decided to use the four-tiered cancer somatic interpretation framework rather than the ACMG germline variant reporting framework, as the ACMG framework was designed for monogenic or Mendelian variants for rare variants with high impact. We focus almost exclusively on non-syndromic autism patients, such that the genetic contribution is likely polygenic and has a low to moderate impact, and the likelihood of detecting classic Mendelian germline variants would be low [30]. Therefore, we decide to adapt the more flexible cancer somatic interpretation framework and integrate phenotypic/genotypic data for inference of pathogenicity. It is not surprising that Tier 1 variants were rarely found, since none of the patients in our cohort were diagnosed with syndromic autism. We decided that one variant could qualify for the Tier 1 category, which is a splice site variant in *SLC12A5* encoding for KCC2 chloride/potassium cotransporter. In addition, the reported highly prevalent medical and psychiatric co-morbid conditions are also important to identify and address.

Finally, we provide concrete “next steps” for further specific testing, targeted treatment trials, and preventive care guidance for patients and families, which is an element that is recommended by recent literature based on patient feedback, but often missing in DTC genetic reports. With the six trios spanning a variety of age, sex, and disease severities, we provided preliminary evidence that a WGS-based genetic reporting approach can be informative to understand the genetic basis of the ASD pathophysiology and comorbidities in non-syndromic ASD patients as well as in providing targeted care. In accordance with genetic reporting “best practices,” we highlight the “actions to be taken,” provide patients with all technical information, use visual display of information, and cite primary sources of information and support [31]. Despite the advantages of this reporting pipeline, we recognize that full clinical implementation will depend on further validation in a prospective cohort and/or validation using publicly available WGS datasets.

Nearly one-quarter of the parents of children with ASD in the United States have taken their affected children to undergo genetic testing [15]. Many parents expressed their interest in a full return of results regardless of medical implications. In particular, parents are calling for performing molecular tests that could be available earlier in development such that early intervention with diagnosis and intervention could be possible [16]. While each patients’ experience with the clinical testing are generally positive with adequate follow up from the patients’ treating physicians, the majority of existing DTC genetic reports are vague, difficult to understand, and barely offer meaningful interpretations of its clinical significance. In contrast to DTC, we provide full disclosure of the bioinformatics pipeline, methodologies, and offer parents/clinicians the opportunity for re-analysis as the knowledgebase and databases rapidly evolve. For example, several recent, large cohort studies shed new light on genetic risk factors of ASD. The largest WGS study, which included over 5000 participants, identified genetic causes for greater than 10% of ASD cases, while the largest WES study, with nearly 12,000 ASD cases, detected 102 risk genes [13,14]. The largest genome-wide association study based on SNP arrays most recently identified five genetic loci that are highly associated with ASD using a sample of more than 18,000 cases [12].

Beyond the first-line microarray-based genetic testing, genetic testing for patients with ASD remains controversial. Currently, WGS or WES based genetic testing is still rare, even in leading academic clinical laboratory settings. Geisinger and University of Washington in St. Louis are among the only two clinical laboratories that routinely offer WGS to patients diagnosed with ASD.

WGS holds great promise to facilitate personalized diagnosis and management for patients with ASD and is likely going to be a more effective genetic testing approach for ASD in the future in comparison to microarray-based or panel-based testing that dominate the ASD genetic testing landscape today [32,33]. For panel-based testing, the set of genes included requires continual revision, and reanalysis is limited. In fact, WES has already been recommended as a first-tier clinical test for individuals with unexplained NDDs by a multidisciplinary expert group following completion of recent scoping review and meta-analysis of diagnostic yield [34]. As our knowledge and understanding of NDDs expand, a comprehensive genomic analysis may offer greater flexibility for reanalyzing the data. At the current early stage of using WGS for clinical applications, the precise fraction of individuals who might benefit from sequencing for disease prevention or early diagnosis remains uncertain even in the general population; however, the “diagnostic yield” will likely change over time [35].

We also acknowledge the limitations of our approach. First, WGS and in-depth reporting is expensive and labor intensive as it requires highly trained professionals, thus limiting its scalability. Second, we used a patient cohort with a small sample size in this proof-of-principle study and did not sequence all variants for Sanger confirmation. Third, the cohort only consisted of patients of Asian ethnicity. A larger cohort of ASD patients will provide a better estimation of the diagnostic yield of our WGS platform. Lastly, we had concerns that WGS results with variants of uncertain significance may add to excessive anxiety and worries for patients and families prior to the initiation of the project; however, genetic testing disclosure did not lead to excessive anxiety of the subjects, similar to reports from the literature in our experience.

Looking into the future, we hope to incorporate the identification of intronic/intergenic variants and calculation of polygenic risk score (PRS) with our existing WGS framework, which requires further research data to support their clinical relevance. One major critique of a WGS approach is the lack of a reliable method to analyze risk contribution in intronic/intergenic regions. This may change with the flourishing interest in research in this area [36,37,38]. Additionally, we plan to build further analytic modules for detecting intronic alterations in the near future. The accuracy of PRS is still limited for ASD. Common genetic variation contributes significantly to the risk of ASD, which is estimated to be between 15% and 50% [39,40]. The most recent GWAS on Autism with ~18,000 ASD cases reported five such loci [10]. We expect that future GWAS on ASD would yield much more replicable results that explain a higher percentage of variance. Furthermore, new studies have utilized multi-omics approaches to integrate patients’ gene mutations and expression data to better understand the genetic contribution to ASD. Based on our interaction with the patients and their families, gene expression data is of great interest to many of them, and we will attempt to incorporate this approach going forward when designing updated interpretation pipeline [41,42].

## Figures and Tables

**Table 1:** Manual review of variants in known, well-established ASD risk genes or chromosomal region deletion/duplication.

Gene		Chromosomal Regions
*AHI1*	*NLGN4X*	15q11-13 maternal deletion
*CACNA1C*	*PTEN*	15q11-13 duplication
*CHD8*	*SHANK3*	15q11-13 paternal deletion
*CNTNAP2*	*TSC1*	15q11-13 duplication
*EIF4E*	*TSC2*	1q21.1
*FMR1*	*UBE3A*	7q11.23
*FMR1-AS1*	*GABAr*	16p11.2
*GBX2*	*PCKB1*	18q12.1
*KIF1A*	*DCHR7*	22q11.2
*MECP2*	*OXTR*	22q13
*NF1*	*SLC6A4*	17p11.2
*NLGN3*	*FOLR1*	
*KIZ*	*XRN2*	

**Table 2:** Overview of subject demographics and characteristics.

Subject	Age (Year)	BMI	Sex	ASD Severity	Comorbidities
#1	6.6	23.6	Female	Low	Hyperactivity, emotional lability, aggressive/destructive behavior, self-injury, sleep disturbance, premature birth, autoimmunity, allergic rhinitis, eczema, leaky bowel, and GI disturbance
#2	18.1	18.1	Male	Low	Allergic rhinitis, sinusitis, autoimmunity, erosive ileitis, H.Pylori/GERD; Developed seizures 1 year after this genetic testing
#3	19.6	26.7	Female	Moderate	OCD, Anxiety, Allergic rhinitis, autoimmunity
#4	6.9	16.5	Male	High	Allergic rhinitis, GI disturbance, emotional lability
#5	5.7	16.0	Male	Moderate	Hyperactive, emotional lability, sleep disturbance, allergic rhinitis, eczema, GERD
#6	3.5	16.1	Male	Low	Hyperactive, emotional lability, aggressive/destructive behavior, sleep disturbance, eczema, anemia, autoimmunity, allergic rhinitis, GI disturbance

**Table 3:** Summary of all variants and results of Sanger sequencing in one participant (subject #1).

Gene Name and Location	Variant Type	Annotation on ClinVar or SFARI	Encoded Protein	Signaling Pathways/Neuronal Circuitry	Other Conditions Associated with the Variant	Mutation Confirmation by Sanger
*SLC12A5* (chr20:46022977:A:G)	Splice region variant	SFARI risk gene	KCC2 (Type 2 K^+^-Cl^−^ cotransporter)	Enhancement of the NF-κB/MMP-7 signaling pathway; Glycinergic signaling pathway. Regulates neuronal excitability	Epileptic encephalopathy, early infantile	Yes
*IER3IP1* (chr18:47156119:A:G)	Splice region variant	Pathogenic on ClinVar	IER3IP1	Although highly expressed in the brain, its role in the CNS circuitry is unknown	Microcephaly, epilepsy, diabetes syndrome	N.A.
*AIFM1* (chrX:130136710:T:C)	3 prime UTR	Likely pathogenic on ClinVar	Mitochondrial flavin adenine dinucleotide-dependent oxidoreductase	Ceramide signaling pathway, innate immunity; has known expression and functionsin the CNS	Mitochondrial encephalopathy	No
*CEP19* in 3q29 (chr3:196711952:A:G)	SNV	SFARI risk gene	Centrosomal Protein 19	Ciliary entry of intraflagellar transport; Although highly expressed in the brain, its role in the CNS circuitry is unknown	Delayed development (speech delay), mild or moderate intellectual disability, gastrointestinal disorders, morbid obesity	N.A.

## Data Availability

Raw sequencing data were generated at Novogene USA. The full raw sequencing data are not publicly available due to restrictions (e.g., their containing information that could compromise the privacy of research participants). The data that support the findings of this study (e.g., a small chunk of the genomic data for a specific individual that harbors the reported variant) are available on request from the corresponding author following approval by the IRB for raw data sharing. The data are not publicly available due to institutional restrictions on the public access of new human genetic sequence variants.

## Supplementary Materials

The following are available online at https://www.mdpi.com/2073-4409/11/1/10/s1, Figure S1: Average sequencing depth (bar plot) and coverage (dot-line plot) in each chromosome. The *x*-axis represents chromosome; the left *y*-axis is the average depth; the right *y*-axis is the coverage (proportion of covered bases); Figure S2: Sample ASD WGS genetics report for participants; Table S1: Sanger sequencing primer information.

## Appendix A

**Table A1:** Summary of all variants and results of Sanger sequencing (includes variants not presented in Table 3).

Subject	Scale of Variant	Gene Name and Location	Variant Type	Encoded Protein	Signaling Pathways/Neuronal Circuitry	Other Conditions Associated with the Variant	Mutation Confirmed by Sanger?
#2	Small	*GJB2* (chr13:20189473:C:T)	Missense	Connexin 26, CX26 (gap junction protein, beta 2)	Calcium Signaling Pathway	Hearing impairment; Keratitis-ichthyosis-deafness syndrome, autosomal dominant; Mutilating keratoderma; genetic deafness	Yes
#2	Small	*PROKR2* (chr20:5302662:C:G)	Missense	Prokineticin receptor 2	Mood regulation; Gonadotropin-releasing hormone; Neutrophil dependent inflammation; Hyper nociception; Migration of nerve cells; Neurogenesis	Kallmann syndrome 3	No
#2	Large	*PWRN1* in 15q11.2 (chr15:24521630:A:G)	Deletion-Duplication	N.A.	N.A.	N.A.	N.A.
#3	Small	*PROP1* (chr5:177995888:G:A)	Stop gained	Paired-like homeodomain transcription factor	Retinoic acid production and signaling pathway; Regulates neuronal excitability	Pituitary hormone deficiency, combined	Yes
#3	Small	*CYP11B1* (chr8:142874995:G:A)	Missense	Enzyme: 11-beta-hydroxylase	Non identified	Adrenal hyperplasia; congenital Hyperaldosteronism, familial, type I	Yes
#3	Small	*MRE11* (chr11:94459461:G:A)	Stop gained	Double Strand Break Repair Nuclease	DNA damage signaling; Chromatin stability	Hereditary cancer-predisposing syndrome; Ataxia-telangiectasia-like disorder 1	N.A.
#3	Large	*POLR3E* in 16p12.1 (chr16:22305383:GA:G)	Deletion-Duplication	DNA-directed RNA polymerase III subunit RPC5	RNA Polymerase III Transcription Initiation; Transcription of tRNA. May be important for fighting CNS viral infection	N.A.	N.A.
#4	Small	*SLC7A14* (chr3:170480891:C:A)	Missense	Glycosylated, cationic amino acid transporter protein with 14 transmembrane domains	Full-length 771-amino acid SLC7A14 protein has 14 transmembrane domains and an N-glycosylation site in extracellular loop-2	Retinitis pigmentosa	Yes
#4	Small	*GJB2* (chr13:20189473:C:T)	Missense	Connexin 26, CX26 (gap junction protein, beta 2)	Calcium Signaling Pathway	Hearing impairment	Yes
#4	Small	*TXNL4A* (chr11:94459461:G:A)	Intron	DIM1, U5 snRNP-SPECIFIC PROTEIN, a member of the U5 small ribonucleoprotein particle (snRNP)	Spliceosome pathway	Burn-McKeown syndrome	N.A.
#5	Small	*USH2A* (chr1:216078088:C:T)	Splice donor	Usherin	USH protein network pathway	Usher syndrome, type 2A; Retinitis pigmentosa 39	N.A.
#5	Small	*SERPINB7* (chr18:63798670:C:CT)	Frameshift	SERPINB7	Degradation of SERPINB7 protein by 26S proteasome-mediated pathway	Palmoplantar keratoderma, nagashima type	Yes
#5	Small	*BSCL2* (chr11:62692671:C:A)	Missense & NMD transcript	Seipin	Critical in pathway of adipogenesis; Affect neurogenesis in hypothalamus; May be involved in hypothalamic pituitary gland axis function	Charcot-Marie-Tooth disease, type 2; Congenital generalized lipodystrophy type 2	No
#5	Large	*MRNIP* in 5q35 (chr5:179858784:G:A)	Duplication	MRN- interacting protein		N.A.	N.A.
#6	Small	*MYOC* (chr1:171652476:G:A)	Splice region variant & intron variant & NMD transcript variant	Myocilin	Modulator of Wnt signaling pathway; Wild-type MYOC inhibits activation of the IL-1/NF-κB pathway	Glaucoma	Yes
#6	Small	*SLCO1B1* (chr12:21196975:C:T)	Stop gained	Organic anion transporting polypeptide 1B1	Liver-specific member of the organic anion transporter family	Gilbert syndrome; Rotor syndrome	No

**Table A2:** Variants with parental origins versus de novo mutations based on Sanger sequencing.

Gene Name	Modification	Mutation Patient	Mutation Father	Mutation Mother
SLC12A5	chr20:46022977:A:G	yes	no	no
AIFM1	chrX:130136710:T:C	no	yes	no
PROP1	chr5:177995888:G:A	yes	yes	no
CYP11B1	chr8:142874995:G:A	yes	no	yes
MYOC	chr1:171652476:G:A	yes	no	yes
SLCO1B1	chr12:21196975:C:T	no	no	no
SLC7A14	chr3:170480891:C:A	yes	yes	no
TXNL4A	chr18:79988603:CGCGCGCGCTAGCGCCGTGCGTGCTGACGGCATGT:C	yes	yes	no
BSCL2	chr11:62692671:C:A	no	no	no
SERPINB7	chr18:63798670:C:CT	Mutation present but different than the variant called by pipeline (63798670:TGAATGCT:GGAAAGGG)	no	no
GJB2	chr13:20189473:C:T	yes	no	yes
PROKR2	chr20:5302662:C:G	no	no	no
