# Correction: Huang et al. A Next Generation Sequencing-Based Protocol for Screening of Variants of Concern in Autism Spectrum Disorder. *Cells* 2022, *11*, 10

**DOI:** 10.3390/cells11203276

**Published:** 2022-10-18

**Authors:** Jie Huang, Jun Liu, Ruiyi Tian, Kevin Liu, Patrick Zhuang, Hannah Tayla Sherman, Christoph Budjan, Michelle Fong, Min-Seo Jeong, Xue-Jun Kong

**Affiliations:** 1 Athinoula A. Martinos Center for Biomedical Imaging, Massachusetts General Hospital, Harvard Medical School, Charlestown, MA 02129, USA; 2 Department of Global Health, Peking University School of Public Health, Beijing 100871, China; 3 Brigham Women’s Hospital, Harvard Medical School, Boston, MA 02115, USA; 4 Washington University in St. Louis, St. Louis, MO 63130, USA; 5 Dana Farber Cancer Institute, Harvard Medical School, Boston, MA 02215, USA; 6 Department of Psychiatry, Beth Israel Deaconess Medical Center, Harvard Medical School, Boston, MA 02215, USA

In the original publication [1], there was a mistake in Figure 1 as published. The authors have identified a typo in Figure 1, which details the in-house variant annotation bioinformatics pipeline that was applied to screen for variants of concern. In particular, the last step of the bioinformatics pipeline should be labeled as, “#6. Filter by #3, #4, #5”, instead of “Filter by #4, #5, #6”. The existing typo suggests an incorrect order of performing the variant annotation through our in-house bioinformatics pipeline, as the typo shifted the label numbers by 1. The corrected Figure 1 appears below.

**Figure 1:**
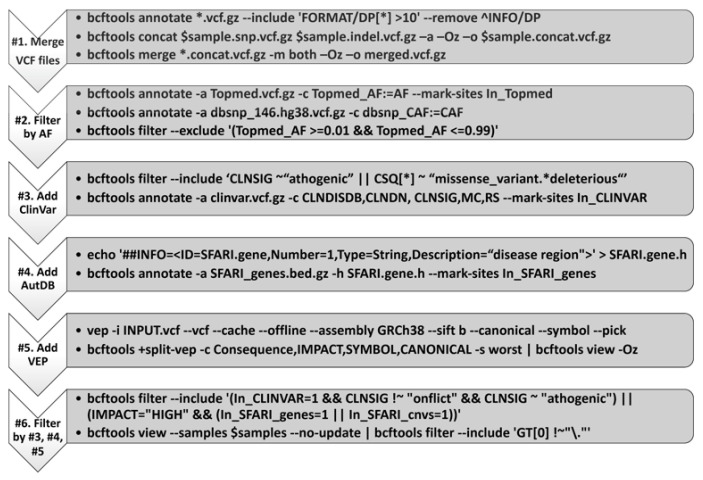
In-house variant annotation pipeline. VCF, variant call format; AF, allele frequency; ClinVar, a freely available, public archive of the relationships among human genetic variants and phenotypes (https://www.ncbi.nlm.nih.gov/clinvar/, accessed on 10 May 2020); VEP, ensemble variant effect predictor (ensemble release 105; https://uswest.ensembl.org/info/docs/tools/vep/index.html, accessed on 10 May 2020). Please note that a number following “#” refers to a step illustrated within the diagram.

The authors apologize for any inconvenience caused. The academic editor has checked this correction and state that the scientific conclusions are unaffected. The original publication has also been updated.
