# Supplementary material for: A Next Generation Sequencing-Based Protocol for Screening of Variants of Concern in Autism Spectrum Disorder
Source: Cells. 2021 Dec 21;11(1):10. doi: 10.3390/cells11010010 (PMC8750892; doi:10.3390/cells11010010)
Supplement: Supplementary file 1 [file cells-11-00010-s001.zip › cells-1413545-supplementary/Figure_S2.pdf]

ASD Genetic Testing Report

1. Subject Information and Plan Details

Subject ID	XXXX	Patient Name	XXXX
		Gender	Female
Subject ID	XXXX	Relationship	Mother
		Gender	Female
Subject ID	XXXX	Relationship	Father
		Gender	Male

Plan Details and Patient Summary

XXXX and both parents participated in this genetic testing process. XXXX was diagnosed with ASD. Both of her parents did not show symptoms for ASD. This ASD genetic testing report is provided with her parents' genome data used as a reference for variants comparison. Autism and medical/psychiatric comorbidity-related genetic variants are evaluated. Patient's pertinent medical/psychiatric history include: hyperactivity, emotional lability, aggressive/destructive behavior, self-injury behaviors, sleep disturbance, autoimmunity, allergic rhinitis, eczema, leaky bowel and GI disturbance; premature birth

Age (yr)	Weight (lb)	Height (inch)	BMI	BMI Percentile	Gender	Race	ASD Severity
XXXX	XXXX	XXXX	XXXX	XXXX	Female	XXXX	XXXX

2. WGS Results

2.1. Small-Scale Mutation (Point Mutation, Substitution, Small Insertion and Deletion)

Gene Name [Location]	Type	Origin	Hereditary	Autism Relevance	Other Conditions	Patient Gene	Father Gene	Mother Gene
**SLC12A5 (chr20:46022975:GA:G) (chr20:46022978:GAGGAGGAGGAGGAA:G)**	Frameshift	Mother	Very likely AD	Strong	Epileptic encephalopathy, early infantile	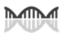	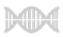	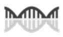
**IER3IP1 (chr18:47156119:A:G)**	Splice region variant & 3 prime UTR variant & NMD transcript variant	Father	Very likely AR	n/a	Microcephaly, epilepsy, diabetes syndrome	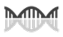	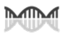	
**AIFM1 (chrX:130136710:T:C)**	3 prime UTR variant & NMD transcript variant	Father	XL	n/a	Deafness	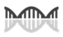	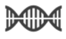	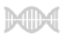

2.2. Large-Scale Mutation (Copy Number Variants, Duplication/Deletion, Chromosomal Inversions)

Gene Name [Location]	Type	Origin	Hereditary	Autism Relevance	Other Conditions	Patient Gene	Father Gene	Mother Gene
**CEP19 in 3q29 (chr3:196711952:A:G)**	Deletion-Duplication	Mother	Very likely AR	Increased risk	Delayed development (speech delay), mild or moderate intellectual disability, gastrointestinal disorders, morbid obesity, spermatogenic failure	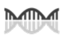		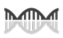

3. **Result Analysis**

3.1. ASD-relevant gene: SLC12A5 (Tier 1 variant)

General Information			
**Gene name**	Encoded protein	Protein Function	Expression patterns<br>(i.e. which organs and tissues are they expressed)
**SLC12A5 (solute carrier)**	KCC2<br>(Type 2 K+-Cl- cotransporter)	KCC2 uses the K+ gradient generated by the N^+-K^+ ATPase pump to extrude Cl- against its electrochemical gradient from neuronal cells (1)<br><br>KCC2 etsablishes an inward l- electrochemical gradient necessary for fast synaptic inhibition (3)<br><br>KCC2 action is crucial for the inhibitory effects of GABA and glycine in most synaptic circuits (9)	KCC2 expressed cell bodies and dendrites of mature neurons of the CNS (1) (9)<br><br>Expression is downregulated in the setting trauma, hypoxia, seizures, nerve injury and spinal cord injury associated with neuropathic pain (9)<br><br>SLC12A5 expression is also seen in normal colon tissues (8)

Function			
Prediction based on protein structures	In vitro studies	In vivo animal studies	Human studies
- There are critical phosphorylation sites located on the protein itself for regulation purposes (1)		- Knock-in mice showed profound resistance to seizures without altered basal neuronal excitability (1)<br><br>- High expression of SLC12A5 was associated with more aggressive lung adenocarcinoma characteristics and poor prognosis – SLC12A5 may have oncogenetic potential (2)	- Evaluated the expression level of KCC2 in polymorphonuclear cells and their correlation with microstructural abnormalities – MRI analysis was used – molecular studies showed significant decrease in KCC2 in epilepsy patients – downregulation of KCC2 and microstructural abnormalities might contribute to observed refractoriness in temporal lobe epilepsy (5)

Pathways involved		
Signaling pathway	Metabolic pathway	Neuronal circuitry
- SLC12A5 has possible oncogenic effects. (6)<br>- Involved in enhancing the NF-κB/MMP-7 signaling pathway – SLC12A5 promotes tumor invasion/metastasis of bladder urothelial carcinoma through enhancing this pathway (6)<br>- Glycinergic signaling pathway (9)	- Chloride homeostasis – when KCC2 is dysfunctional, there's exaggerated Cl- extrusion (1)	- Regulates neuronal excitability – KCC2 dysfunction can lead to neuronal hyperexcitability – leading to epilepsy (1)

Experimental therapies	
FDA approved medications	Other potential medications
- Bumetanide is a NKCC1 blocker which decrease intracellular chloride concentration (achieving the same goal as KCC2 activator). It is FDA approved the treatment of edema associated with congestive heart failure, hepatic and renal disease, including the nephrotic syndrome.<br>- Multiple trials in autism patients show promising results. Generally well tolerated by pediatric patients.	- Tang et al (Science Translational Medicine, 2019) validated a number of drugs (such as FDA approved anti-tumor kinase inhibitors) and supplements (such as resveratrol, piperine and indirubin) that can significantly alter the expression of KCC2 protein in neurons, and reverse autism behaviors in a Rett syndrome mouse model. A detailed discussion of these potential medications will be provided during consultation.<br>- Multiple small molecules show binding and possible pharmacological action to modulate functions of KCC2 through our virtual drug screen platform, but their precise roles have not been studied. Details of these drugs can be requested upon consultation.

References

1) https://www.ncbi.nlm.nih.gov/pmc/articles/PMC6873151/pdf/fncel-13-00515.pdf
2) https://www.ncbi.nlm.nih.gov/pmc/articles/PMC6811597/pdf/41389_2019_Article_167.pdf
3) https://sci-hub.tw/10.1126/scisignal.aaw9315
4) https://www.ncbi.nlm.nih.gov/pmc/articles/PMC6420604/pdf/41467_2019_Article_8933.pdf
5) https://sci-hub.tw/10.1097/wnr.0000000000001216
6) https://www.ncbi.nlm.nih.gov/pmc/articles/PMC5386524/pdf/cddis2017118a.pdf
7) http://www.jbc.org/content/286/35/30492.full.pdf?with-ds=yes
8) https://www.nature.com/articles/cr201443
9) https://sci-hub.tw/10.1212/WNL.0b013e318283bb1c

3.2. ASD-relevant gene: AIFM1 (Tier 3 variant)

General Information			
**Gene name**	Encoded protein	Protein Function	Expression patterns (i.e. which organs and tissues are they expressed)
**AIFM1 (Apoptosis Inducing Factor Mitochondria Associated 1)**	Mitochondrial flavin adenine dinucleotide (FAD)-dependent oxidoreductase (1)	Oxidative phosphorylation (OxPhos), redox control and program cell death (1)	Its RNA and protein expression is found in all tissues (11).

Function			
Prediction based on protein structures	In vitro studies	In vivo animal studies	Human studies
	- Role in apoptosis and cell death (2)	- AIFM1 gene function was characterized in mouse auditory function (inner ear). (4)<br><br>- AIF serves as a free radical scavenger. (8)<br><br>- Loss of AIF function causes abnormal cell death presumably because of reduced mitochondrial respiratory chain complex 1 activity. (9)	- Human AIF is a mitochondrion-specific protein that binds FAD and attaches by an N-terminal transmembrane domain to the inner mitochondrial membrane but it functions as an NADH oxidase. After mitochondrial import, cleavage results in the mature protein (3)

Pathways involved		
Signaling pathway	Metabolic pathway	Neuronal circuitry
- Ceramide signaling pathway, innate immunity.	- Apoptotic Pathways in Synovial Fibroblasts<br>- Chromatin condensation and large-scale DNA degradation<br>- Caspase-independent pathway of programmed cell death that controls early morphogenesis (6)<br>- Necroptosis	- Non-identified

References

1) https://www.omim.org/entry/300169
2) https://sci-hub.tw/https://www.nature.com/articles/17135
3) https://www.cell.com/ajhg/fulltext/S0002-9297(10)00145-X
4) https://jmg.bmj.com/content/jmedgenet/52/8/523.full.pdf
5) https://sci-hub.tw/https://www.nature.com/articles/35069004
6) https://sci-hub.tw/https://www.nature.com/articles/35069004
7) https://sci-hub.tw/https://science.sciencemag.org/content/297/5579/259/tab-pdf
8) https://sci-hub.tw/https://www.nature.com/articles/nature01034
9) https://www.pnas.org/content/pnas/103/26/9918.full.pdf
10) https://www.pnas.org/content/pnas/103/46/17366.full.pdf
11) https://www.proteinatlas.org/ENSG00000156709-AIFM1/tissue

3.3. ASD-relevant gene: CEP19 (Tier 3)

General Information			
**Gene name**	Encoded protein	Protein Function	Expression patterns (i.e. which organs and tissues are they expressed)
**CEP19**	Centrosomal Protein 19	Centrosomal and ciliary protein (4)	Found in many tissues/organs especially the brain, lung, and male organs (testes) (9).

Function			
Prediction based on protein structures	In vitro studies	In vivo animal studies	Human studies
- The C-terminus of CEP19 is required for both its localization to centrioles and for its function in ciliogenesis (6).	- CEP19 CRISPR KO cells are severely impaired in their ability to form cilia (6)	- Homozygous CEP19 KO mice were morbidly obese, hyperphagic, glucose intolerant, and insulin resistant (1).	- Mutation associated with delayed development (speech delay), mild or moderate intellectual disability (10).<br><br>- Loss of function mutation in CEP19 is also linked to ciliopathy phenotypes like morbid obesity and defective sperm motility (3)<br><br>- Gene resides in a region of human chromosome 3 that is linked to morbid obesity. (1)

Pathways involved		
Signaling pathway	Metabolic pathway	Neuronal circuitry
- Ciliary entry of intraflagellar transport (7)	- Possible role in glycemic control due to its association with obesity	- Although highly expressed in the brain, its role in the CNS circuitry is unknown.

References

1) https://www.genecards.org/cgi-bin/carddisp.pl?gene=CEP19
2) https://jcs.biologists.org/content/joces/132/2/jcs224428.full.pdf
3) https://sci-hub.tw/https://www.sciencedirect.com/science/article/pii/S0002929713004825
4) https://sci-hub.tw/https://jmg.bmj.com/content/55/3/189.long
5) https://www.cell.com/developmental-cell/fulltext/S1534-5807(17)30504-X?_returnURL=https%3A%2F%2Flinkinghub.elsevier.com%2Fretrieve%2Fpii%2FS153458071730504X%3Fshowall%3Dtrue
6) https://www.ncbi.nlm.nih.gov/pmc/articles/PMC5493781/pdf/rsob-7-170114.pdf
7) https://www.ncbi.nlm.nih.gov/pmc/articles/PMC5556974/pdf/nihms879340.pdf
8) https://www.ncbi.nlm.nih.gov/pmc/articles/PMC3852924/pdf/main.pdf
9) https://www.proteinatlas.org/ENSG00000174007-CEP19/tissue
10) https://pubmed.ncbi.nlm.nih.gov/29127258/

3.4. Comorbidity related gene: IER3IP1: brief summary

Brief Summary		
Encoded protein	Functions	Other Conditions associated with the variant
IER3IP1 (Immediate Early Response 3 Interacting Protein 1)	May play a role in the ER stress response by mediating cell differentiation and apoptosis. Although highly expressed in the brain, its role in the CNS circuitry is unknown.	epilepsy, diabetes

Conclusion: Patient has multiple mutations in autism risk genes or genes associated with comorbid medical and neuro-psychiatric conditions.

4. Clinical Interpretation

4.3. Mutations that have potential impact on hallmarks of ASD Core pathophysiology

- Mutation in SLC12A5 is associated with decreased function of a chloride channel (KCC2) which is important for the functions of Gaba-ergic neurons.
- Mutation of the gene KCC2 is linked to excitation/inhibition imbalance.
- AIFM1 is broadly expressed in different tissues and important in the ceramide signaling pathway. It is important for cell survival and proliferation, and neuron survival.
- CEP19 mutations have been linked the developmental delay.

Illustrations of the key potential pathogenic pathways (SLC12A5)

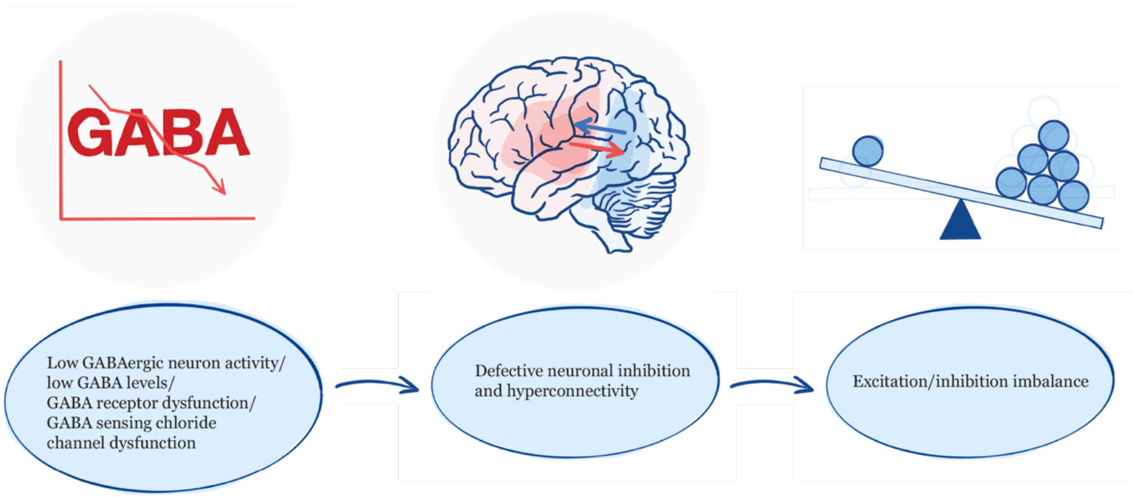

4.4. Mutations that have implications for comorbidities

4.4.1. Seizure

- The mutation of SLC12A5 also confers higher risk of seizure disorders.

4.4.2. Immune dysfunction

- Patient may have deficits in his/her immune function due to AIFM1 mutation.
- Post-infectious autoantibodies against brain tissues, and common gastrointestinal infections can be at increased risk.

4.4.3. Metabolic syndromes

- CEP19 is has possible role in glycemic control due to its association with obesity and IER3IP1 is highly expressed in pancreas so it may also regulate sugar metabolism.

4.4.4. Gastrointestinal disorders: could be implicated

4.4.5. Sleep disorder: could be implicated

4.4.6. Mitochondrial dysfunction: could be implicated

4.4.7. Psychiatric conditions (anxiety, depression, ODD, ADHD): could be implicated and increased at risk.

4.4.8. Others: to be identified

4.5. Recommendations/next steps:

4.5.1. Validations of mutations by Sanger sequencing.

4.5.2. Drug screening completed: Multiple drugs and supplements that may target the protein or the related E/I balance neurological pathways have been identified, could be beneficial for disease/core symptom modification (see Results Analysis 3.1).

4.5.3. Screening EEG to monitor occult seizures. Recommend testing for autonomic dysfunction.

4.5.4. Suggest to closely monitor immune function profile, autoantibodies, infections

4.5.5. Check for metabolic syndrome, may benefit from a low sugar diet.

4.5.6. Please schedule a consultation for further recommendations.

5. **Appendix**

1) Important Declaration

(1) There have been increasing scientific reports demonstrating that certain gene mutation is connected to ASD. Many researchers have discovered the human genome markers potentially related to ASD syndromes. Current examination of ways to treat ASD syndrome and relative disease using knowledge about the human genome is underway. However, whole genome sequence analysis is not yet approved for disease prevention, treatment, and therapy. This report should not be used for disease prevention, diagnosis therapy or cure.

(2) Information provided in this report is limited by ASD research progress and could be updated according to future researches.

(3) All individual identifying information regarding the gene data will be removed so as to maintain personal privacy of health information. We promise never to reveal any personal and individualized genome results without permission.

(4) Due to the technique limitations with Next Generation Sequencing, Sanger sequencing is needed in order to confirm the results from this report.

2) Whole Genome Sequencing
Novogene – 30x coverage
