# Supplementary figures and images for: A Next Generation Sequencing-Based Protocol for Screening of Variants of Concern in Autism Spectrum Disorder
Source: Cells. 2021 Dec 21;11(1):10. doi: 10.3390/cells11010010 (PMC8750892; doi:10.3390/cells11010010)

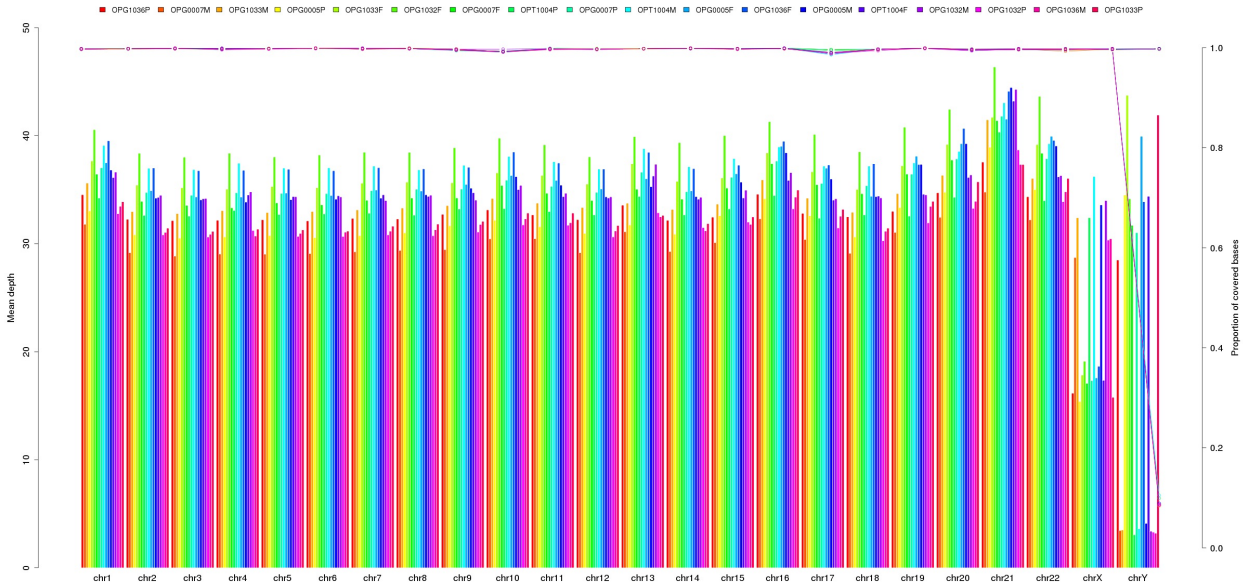

Supplement: Supplementary file 1 [file cells-11-00010-s001.zip › cells-1413545-supplementary/Figure_S1.pdf]
